# Editorial: Harnessing resilient flora for maximized crop efficiency and stress tolerance

**DOI:** 10.3389/fpls.2026.1862220

**Published:** 2026-05-07

**Authors:** Tejas C. Bosamia, Kalyani M. Barbadikar, Bhagwat Nawade, Gautam Saripalli

**Affiliations:** 1 Department of Agricultural Biotechnology, Anand Agricultural University, Anand, Gujarat, India; 2 Department of Biotechnology, Indian Council of Agricultural Research-Indian Institute of Rice Research, Hyderabad, India; 3 Hawkesbury Institute for the Environment, Western Sydney University, Richmond, NSW, Australia; 4 Clemson University Pee Dee Research and Education Center, Department of Plant and Environmental Sciences, Florence, SC, United States

**Keywords:** abiotic stress, epigenetics, nanobiotechnology, resilient flora, yield improvement

The growing unpredictability of climate and resource constraints continues to challenge global agriculture, demanding innovative strategies for crop improvement. This Research Topic *“Harnessing Resilient Flora for Maximized Crop Efficiency and Stress Tolerance”* brings together a diverse yet interconnected set of studies that collectively advance our understanding of plant resilience from genetic regulation to field productivity. This Research Topic highlights advances in genomics (epigenetics, transcriptomics) and nanobiotechnology for stress tolerance (mainly abiotic stresses like drought, salinity and nitrogen use efficiency NUE) not only in major cereals like wheat and rice, but also in other plant species like halophytes (mangroves), ornamental plants (sweet osmanthus) and bioenergy crops.

## Genetics, nanobiotechnology and molecular aspects of abiotic stress tolerance

In cereals, optimum application of nitrogen fertilizers plays a significant role in enhancing grain micronutrient composition (especially zinc and iron). Consistent efforts are being made to identify markers linked to the NUE associated traits which could aid in molecular breeding for improved NUE. Malakondaiah et al. identified genomic regions controlling nitrogen use efficiency (NUE) in wheat which can be targeted for breeding purposes. In total, eight important QTLs under nitrogen deficient conditions were identified using biparental mapping. These QTLs were linked to metal concentrations like zinc, calcium, iron, nickel and potassium. Twelve recombinant inbred lines (RILs) were also identified which were superior for multiple traits simultaneously as revealed through multi-trait genotype–ideotype distance index (MGIDI). The study revealed the genetic and molecular basis of micronutrient accumulation under nitrogen deficient conditions in wheat and identified important genomic and genetic resources for breeding wheat for improved NUE.

Drought is another important abiotic stress which is responsible for significant yield losses in major cereals. A recent news report suggests a reduction of 13% in grain yields due to climate change, especially severe dry and warmer conditions in last 50 years (https://resoilfoundation.org/en/agricultural-industry/climate-grain-yields-reduced/). Elsherif et al. used a nanobiotechnology-based approach to enhance drought tolerance in barley. The authors-imposed drought stress on barley plants under pot conditions by irrigating at 30% field capacity. After drought stress, the plants were treated with biogenic calcium phosphate nanoparticles. These plants showed significant improvement in growth and plant functions despite drought stress, not only in morphophysiological parameters but also in molecular responses as revealed through gene expression studies. Altogether, their study explored a novel approach for enhancing drought tolerance using nanobiology-based tools.

The impact of salt stress was studied in rice and a mangrove species by Xu et al. and Wang et al. respectively. In rice, Xu et al. examined the impact of mixed oligosaccharides on the growth of rice seedlings under salinity (100 mmol L^−1^ NaCl) and alkalinity (10 mmol L^−1^ Na_2_CO_3_) stresses. Significant improvements in growth, physiology, and root morphology were observed following oligosaccharide priming. RNAseq analysis identified differential gene expression patterns associated to photosynthesis under salt stress conditions, thus revealing the enhancement of photosynthesis and mitigated oxidative damage.

In mangrove tree species (*Avicennia marina*), Wang et al. identified an endogenous promoter, *AMGT1P33*, and examined its role in driving the expression of genes for enhanced salt tolerance in *Arabidopsis* through GUS assay. Their findings suggest that such regulatory elements from extremophytes can be effectively utilized in crop systems to elucidate and enhance salt tolerance mechanisms, thereby facilitating the development of transgenic approaches for functional validation and improvement of salinity tolerance in important crop species.

## Genetics of yield improvement in cereals

Yield is a complex trait controlled by many genes/QTL and therefore, studying the genetics of yield improvement has always been challenging for breeders and geneticists. Total yield is determined by a number of associated traits like grain number, grain size, tiller number, plant architecture. etc. Chachar et al. compiled literature focused on genetics and molecular aspects of tiller development and yield improvement in the form of a comprehensive review. Their detailed literature survey revealed the involvement of complex genetic, molecular and environmental interactions in controlling grain yield in cereals. Available GWAS and biparental mapping studies were deeply reviewed for pinpointing the genomic regions controlling tiller development. By combining transcriptomic studies with genetic mapping, some important genes like *TaMAX1, TaMOC1*, and *TN1 in wheat, ZmTB1, ZmD14, and ZmMOC1* in maize, along with *MAX1*-like genes, *OsMAX1, and OsHAM2* in rice were highlighted along with phytohormonal regulation. As tillering directly influences crop productivity, advances in this area hold significant promise for sustainable yield enhancement.

## Epigenetics and crop improvement

Epigenetic regulation, encompassing DNA methylation, histone modifications, and non-coding RNAs, orchestrate dynamic changes in chromatin architecture without altering the underlying DNA sequence. Epigenetic memory enables priming responses to recurrent environmental cues, thereby enhancing resilience and nutrient use efficiency.

Vela and Steadman, reviewed in detail the progress made and research gaps in the epigenetic research in bioenergy crops. Zhang et al. studied the spatio-temporal tissue specific expression of important microRNAs (based on miRNA sequencing) in *Osmanthus fragrans* under different abiotic stresses (cold, salinity and drought), hormonal and metal ion treatments in different tissues to examine their suitability as candidate reference genes. This study provides the first validated set of reference miRNAs for accurate normalization in miRNA expression analyses under abiotic stress and developmental conditions in *O. fragrans*.

Together, these articles exemplify a multidimensional approach to crop resilience leveraging natural diversity, refining molecular tools, and integrating cutting-edge technologies. This Research Topic not only expands the scientific foundation of stress biology but also provides insights for future breeding and biotechnological interventions. As agriculture moves toward sustainability under changing climates, such integrative efforts will be pivotal in shaping the next generation of resilient and efficient cropping systems.

